# Longitudinal comparisons of mental health, burnout and well-being in patient-facing, non-patient-facing healthcare professionals and non-healthcare professionals during the COVID-19 pandemic: findings from the CoPE-HCP study

**DOI:** 10.1192/bjo.2022.579

**Published:** 2022-09-27

**Authors:** Vikas Kapil, George Collett, Thomas Godec, Jaya Gupta, Carmela Maniero, Sher M. Ng, Iris McIntosh, Abhishek Kumar, Satheesh Nair, Ashish Kotecha, Azara Janmohamed, Sotiris Antoniou, Rehan Khan, Mohammed Y. Khanji, Imrana Siddiqui, Ajay Gupta

**Affiliations:** Barts Heart Centre, St. Bartholomew's Hospital, Barts Health NHS Trust, UK; William Harvey Research Institute, Queen Mary University of London, UK; and Department of Clinical Pharmacology, The Royal London Hospital, Barts Health NHS Trust, UK; William Harvey Research Institute, Queen Mary University of London, UK; Child and Adolescent Mental Health Service, South West London and St George's Mental Health NHS Trust, UK; Barts Heart Centre, St. Bartholomew's Hospital, Barts Health NHS Trust, UK; and William Harvey Research Institute, Queen Mary University of London, UK; Barts Heart Centre, St. Bartholomew's Hospital, Barts Health NHS Trust, UK; Islington Learning Disability Partnership, Camden & Islington Foundation Trust, UK; Department of Cardiology, Wrightington, Wigan and Leigh NHS Foundation Trust, UK; Department of Cardiology, Glan Clwyd Hospital, Betsi Cadwaladr University Health Board, UK; Department of Cardiology, Royal Devon and Exeter Hospital, UK; Department of Clinical Pharmacology, St George's University Hospitals NHS Foundation Trust, UK; Barts Heart Centre, St. Bartholomew's Hospital, Barts Health NHS Trust, UK; and Cardiovascular Health, UCLPartners, UK; Department of Obstetrics and Gynaecology, The Royal London Hospital, Barts Health NHS Trust, UK; Barts Heart Centre, St. Bartholomew's Hospital, Barts Health NHS Trust, UK; William Harvey Research Institute, Queen Mary University of London, UK; Cardiovascular Health, UCLPartners, UK; and Department of Cardiology, Newham University Hospital, Barts Health NHS Trust, UK; Wellbeing Hub, Newham Training Hub, UK; NHS North East London Integrated Care Board (ICB), UK; and Woodgrange Medical Practice, UK

**Keywords:** Burnout, Mental health, COVID-19, Epidemiology, Healthcare professionals

## Abstract

**Background:**

The COVID-19 pandemic may disproportionately affect the mental health of healthcare professionals (HCPs), especially patient-facing HCPs.

**Aims:**

To longitudinally examine mental health in HCPs versus non-HCPs, and patient-facing HCPs versus non-patient-facing HCPs.

**Method:**

Online surveys were distributed to a cohort at three phases (baseline, July to September 2020; phase 2, 6 weeks post-baseline; phase 3, 4 months post-baseline). Each survey contained validated assessments for depression, anxiety, insomnia, burnout and well-being. For each outcome, we conducted mixed-effects logistic regression models (adjusted for *a priori* confounders) comparing the risk in different groups at each phase.

**Results:**

A total of 1574 HCPs and 147 non-HCPs completed the baseline survey. Although there were generally higher rates of various probable mental health issues among HCPs versus non-HCPs at each phase, there was no significant difference, except that HCPs had 2.5-fold increased risk of burnout at phase 2 (emotional exhaustion: odds ratio 2.50, 95% CI 1.15–5.46, *P* = 0.021), which increased at phase 3 (emotional exhaustion: odds ratio 3.32, 95% CI 1.40–7.87, *P* = 0.006; depersonalisation: odds ratio 3.29, 95% CI 1.12–9.71, *P* = 0.031). At baseline, patient-facing HCPs (versus non-patient-facing HCPs) had a five-fold increased risk of depersonalisation (odds ratio 5.02, 95% CI 1.65–15.26, *P* = 0.004), with no significant difference in the risk for other outcomes. The difference in depersonalisation reduced over time, but patient-facing HCPs still had a 2.7-fold increased risk of emotional exhaustion (odds ratio 2.74, 95% CI 1.28–5.85, *P* = 0.009) by phase 3.

**Conclusions:**

The COVID-19 pandemic had a huge impact on the mental health and well-being of both HCPs and non-HCPs, but there is disproportionately higher burnout among HCPs, particularly patient-facing HCPs.

The COVID-19 pandemic has had a considerable impact on the mental health of the general population.^1^ However, there is also concern that the mental health of healthcare professionals (HCPs) has been disproportionately affected^2–4^ because of the stress related to caring and working with patients with COVID-19,^5–8^ increased exposure to COVID-19, concern regarding infecting family members,^9–11^ and other unique stressors such as moral injury^12^ and stigma.^11^ This is likely in addition to the mental health impact related to the growing economic insecurity^13^ and financial problems^14^ faced by the general public, and issues such as staff shortages resulting from cuts to public health services in the UK. The mental health impact is likely to result in increased work absences and significant attrition in some job roles, thus it is a priority to broadly understand the impact, dimensions and severity of the COVID-19 pandemic on the mental health of HCPs.^9^

Nonetheless, there is conflicting data regarding the relative impact on the mental health of front-line HCPs (those who work with patients) compared with ‘non-front-line’ HCPs, or HCPs compared with non-HCPs, during this pandemic.^15–18^ Largely these studies have been cross-sectional only,^2,17–19^ or, in the case of the few longitudinal studies, have not repeatedly sampled the same population,^20^ thereby limiting our understanding of the evolution of mental health changes throughout the pandemic. Moreover, although there has been great media interest in burnout, this has not been systematically evaluated in the different professional groups described above over time.

## Aims

To address these gaps, we devised the COVID-19 Disease and Physical and Emotional Wellbeing of Health Care Professionals (CoPE-HCP) study^21^ as an international, observational cohort study assessing mental health, well-being and burnout in HCPs and non-HCPs across three distinct phases for evaluation of multiple domains over time.

This study aimed to examine the risk of probable mental health issues (i.e. the presence of probable depression, anxiety and insomnia), including burnout, in HCPs compared with non-HCPs, as well as patient-facing HCPs compared with non-patient-facing HCPs. We hypothesised that HCPs would exhibit higher rates of these mental health and burnout outcomes compared with non-HCPs, and this would similarly be true when comparing those in patient-facing roles with non-patient-facing, because of the unique pressures faced, such as overwhelmed healthcare systems, lack of effective treatments and lack of effective vaccines (during the initial phase of the COVID-19 pandemic from July 2020 to January 2021).^21^

## Method

The protocol for this study and the broader CoPE-HCP project has already been published.^21^

### Participants

**Fig. 1:**
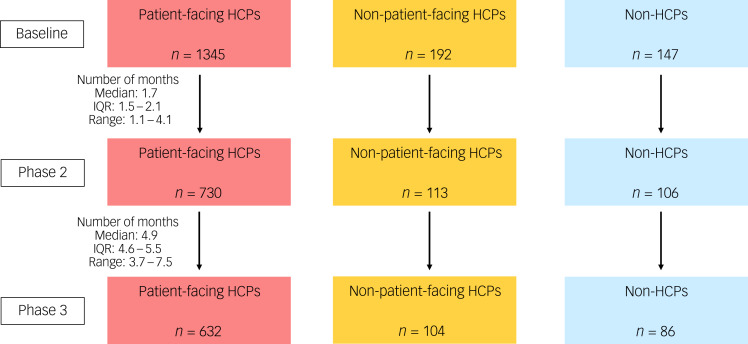
Flow diagram for participant numbers. Total numbers of participants at baseline, phase 2 and phase 3 was 1721, 957 and 830, respectively. These numbers are higher than those included in the figure because not all HCPs could be accurately categorised as patient-facing or non-patient-facing HCPs (e.g. the total number of participants at baseline where patient-facing status and HCP status could be identified is 1713). HCP, healthcare professional; IQR, interquartile range.

The study involved three groups of participants (Fig. 1): patient-facing HCPs (HCPs working with patients with confirmed or suspected COVID-19); non-patient-facing HCPs (HCPs in non-patient-facing roles, not directly in contact with patients confirmed or suspected as having COVID-19); and non-HCPs (non-healthcare academic and research staff of Queen Mary University of London, and other professionals not working with patients confirmed or suspected as having COVID-19).

### Ethical approval and consent

The authors assert that all procedures contributing to this work comply with the ethical standards of the relevant national and institutional committees on human experimentation and with the Helsinki Declaration of 1975, as revised in 2008. All procedures involving human participants were approved by the Cambridge East, Research Ethics Committee (approval number 20/EE/0166), and corresponding details were registered with Clinicaltrials.gov (identifier NCT04433260). Written (online) informed consent was obtained from all participants.

### Materials

Participant recruitment was facilitated via open invitation on institutional websites and email distribution lists at healthcare facilities (for HCPs) and Queen Mary University of London (for non-HCPs).

The baseline survey (July to September 2020) gathered information on age, gender identity, ethnicity, relationship status, educational attainment, existing diagnosis of physical and mental health conditions, and healthcare role (if any). The baseline survey also included validated screening measures assessing probable major depressive disorder, generalised anxiety disorder, insomnia, burnout (emotional exhaustion and depersonalisation domains) and well-being. At the end of the baseline survey, participants were asked if they would consent to receiving invitations to follow-up surveys.

The survey at phase 2 (6 weeks post-baseline) included the same mental health, burnout and well-being measures. The survey at phase 3 (4 months post-baseline) included the same mental health, burnout and well-being measures, and included items asking about positive tests for COVID-19. Participant re-entry was allowed at phase 3 if they had completed baseline, but not phase 2, assessment.

The validated mental health, burnout and well-being measures asked at all phases were as follows: the nine-item Patient Health Questionnaire (PHQ-9) to measure depression;^22^ the seven-item Generalised Anxiety Disorder (GAD-7) to measure anxiety;^23^ the seven-item Insomnia Severity Index (ISI) to measure clinical insomnia;^24^ burnout was measured with single-item indicators of emotional exhaustion and depersonalisation, abbreviated from the Maslach Burnout Inventory;^25^ and the Short Warwick–Edinburgh Mental Well-Being Scale (SWEMWBS) to measure well-being.^26^

These measures were selected because they are widely used and freely available, allowing comparable rates with similar research elsewhere, and have validated cut-off points.

### Statistical analysis

The statistical analysis was conducted with Stata version 17.0 for Windows. Descriptive statistics for sample sociodemographic and baseline characteristics were calculated as frequencies and percentages. Included in the analysis were individuals who could be identified as HCPs or non-HCPs and, if identified as an HCP, could be further identified as patient-facing or non-patient-facing HCPs based on the baseline evaluation. Only participants who had completed baseline assessment and at least one follow-up phase of assessment were included in all analysis.

Separate mixed-effects logistic regression models (for each outcome) were conducted to relate HCP status and patient-facing HCP status to the presence of probable major depressive disorder, generalised anxiety disorder, clinical insomnia, emotional exhaustion, depersonalisation and average/high well-being at each phase. In these models, non-HCPs served as the reference group to compare with HCPs, whereas non-patient-facing HCPs served as the reference category to compare with patient-facing HCPs. These mixed-effects models were adjusted for age, gender, time since COVID-19 peak, highest level of education, relationship status, number of people living in household, existing diagnosis of a mental health condition and existing diagnosis of a physical health condition. A further inclusion criterion for the mixed-effects analysis was the provision of a completed mental health outcome measure from at least one more assessment (in either of the two subsequent phases).

Validated cut-offs were used for the respective mental health, burnout and well-being measures (since the validated cut-offs assess the severity of symptoms and do not provide clinical diagnosis, we define our outcomes as ‘probable’). A score of 10 or higher on the PHQ-9 and GAD-7 indicates probable major depressive disorder and probable generalised anxiety disorder, respectively.^22,23^ A score of 15 or higher on the ISI indicates probable insomnia.^24^ Regarding burnout measures, a score of 4 or higher indicates probable burnout characterised by emotional exhaustion or depersonalisation for both respective scales.^25^ A score of 21 or above on the SWEMWBS indicates average-to-high well-being.^26^

## Results

Of 2100 participants who responded to the online survey, 1721 participants (1574 HCPs and 147 non-HCPs with information on their professional role) were eligible for the longitudinal follow-up. This cohort was further followed up by two more surveys, which were 6 weeks (phase 2; *n* = 957; 851 HCPs and 106 non-HCPs) and approximately 4 months (phase 3; *n* = 830; 744 HCPs and 86 non-HCPs) after the baseline survey (Fig. 1). It must be noted that, of the 830 participants included in phase 3 analysis, a small subsample (*n* = 98) had not completed phase 2 assessment.

To address potential sample biases, the baseline characteristics of those who only responded to the baseline survey (*n* = 666) were mostly similar to those who responded to baseline and at least one follow-up survey (*n* = 1055), except for significant differences in self-defined ethnicity, gender identity and number of people living in the household (Supplementary Table 1 available at https://doi.org/10.1192/bjo.2022.579). Participants who only responded to the baseline survey had relatively higher proportions of self-assigned Asian ethnicity and male gender, and belonged to bigger households (Supplementary Table 1). Mental health outcomes were not significantly different between those who only responded to the baseline survey and those who responded at baseline and least one follow-up survey, according to chi-squared analysis (Supplementary Table 1).

In the UK, July to September 2020 (phase 1) corresponded to the trough of the first wave of COVID-19 and coincided with the easing of the first UK lockdown, as did the follow-up period 6 weeks later (phase 2), but there were increased numbers of COVID-19 cases during phase 2. Phase 3 coincided with the second UK national lockdown during the rise in COVID-19 cases in the winter of 2020.

### Baseline characteristics

At baseline, 1574 (91.5%) were identifiable as HCPs and 147 (8.5%) were non-HCPs (Supplementary Table 2).

Overall, nearly 70% of the participants were older than 35 years, with two-thirds identifying as White (66%) and just under a quarter identifying as Asian (22.3%). Most of the participants were women (70.5%) and lived in households with one more member (86.8%). There were no significant differences in distribution of age, gender identity, ethnicity and family structure between HCPs and non-HCPs. However, the educational attainment (proportion with Master's or PhD) was relatively high among non-HCPs (69.4%) compared with HCPs (39.0%) (*P* < 0.001), primarily because non-HCPs consisted mainly of those employed in the university (academic and research staff).

Within the HCP group, 1537 could be further identified as either patient-facing HCPs (*n* = 1345; 87.5%) or non-patient-facing HCPs (*n* = 192; 12.5%) (Supplementary Table 3). There were no differences in the distribution of gender identity, ethnic group and family structure between patient-facing HCPs and non-patient-facing HCPs, except that non-patient-facing HCPs were significantly older (77.1% aged 36 years or older) than patient-facing HCPs (69.5% aged 36 years or older) (*P* < 0.001) and had relatively lower educational attainment (33.9% had a Bachelor's degree) than patient-facing HCPs (48.9% had a Bachelor's degree) (*P* < 0.001).

Of these, 843 participants (730 patient-facing HCPs and 113 non-patient-facing HCPs) completed phase 2 assessment, and 736 (632 patient-facing HCPs and 104 non-patient-facing HCPs) completed phase 3 assessment. Of the 736 participants included at phase 3, a small subsample (*n* = 93) had not completed phase 2 assessment.

### Evidence of positive COVID-19 test at baseline and follow-up (phase 3)

At study baseline, which corresponded with 6 months into the pandemic, 18.5% of HCPs and 2.8% of non-HCPs reported a positive test for COVID-19. At the phase 3 follow-up, 26.5% of HCPs and 20.0% of non-HCPs reported a positive test for COVID-19 (Supplementary Table 4).

Within the HCPs, 19.1% of patient-facing HCPs and 14.1% of non-patient-facing HCPs reported a positive test for COVID-19 at baseline. At phase 3 follow-up, 26.0% of patient-facing HCPs and 14.4% of non-patient-facing HCPs reported a positive test of COVID-19 (Supplementary Table 5).

### Mental health and burnout among HCPs and non-HCPs

**Fig. 2:**
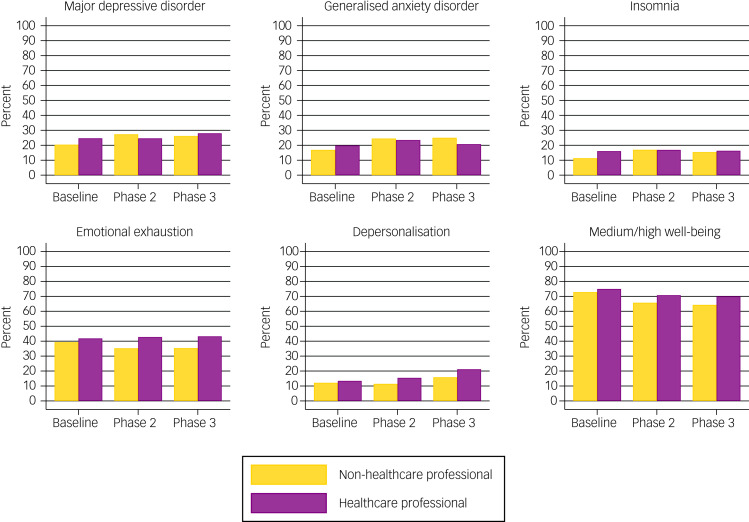
Rates of probable mental health conditions and burnout domains as assessed by validated screening tools in healthcare professionals and non-healthcare professionals at baseline, phase 2 and phase 3.

Figure 2 shows that the rates of probable major depressive disorder, generalised anxiety disorder and clinical insomnia at baseline were generally higher in HCPs than non-HCPs, but did not differ considerably. The subtle difference in the rates of these outcomes continued in the two subsequent follow-ups. At baseline, there was also a higher proportion of HCPs with emotional exhaustion (41.9% of HCPs *v*. 39.3% of non-HCPs) and depersonalisation (13.4% of HCPs *v*. 12.1% of non-HCPs), compared with non-HCPs. At phase 2, 42.8% and 15.5% of HCPs had emotional exhaustion and depersonalisation, respectively, compared with 35.2% and 11.4% of non-HCPs. At phase 3, 43.2% and 21.2% of HCPs had emotional exhaustion and depersonalisation, respectively, compared with 35.4% and 15.9% of non-HCPs. Regarding well-being, a greater proportion of HCPs at each phase (baseline: 75.0%, phase 2: 70.8%, phase 3: 70.0%) met the criteria for average-to-high well-being compared with non-HCPs (baseline: 72.9%, phase 2: 65.7%, phase 3: 64.3%). For specific rates of all outcomes at each phase in HCPs and non-HCPs, see Supplementary Table 6.

**Fig. 3:**
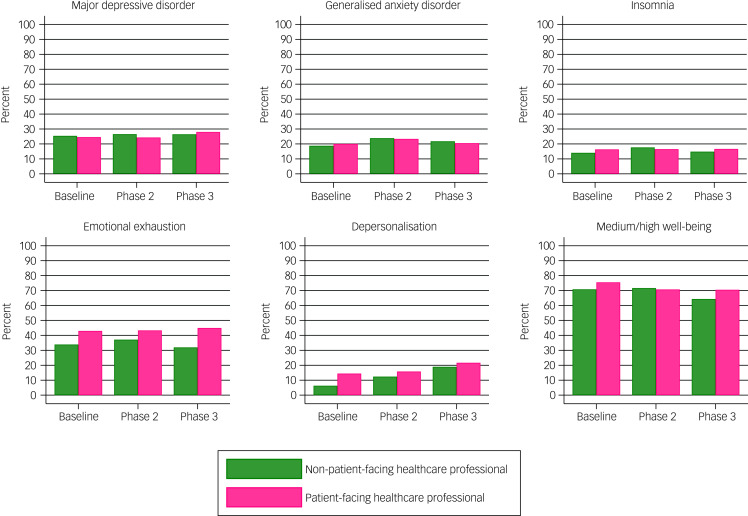
Rates of probable mental health conditions and burnout domains as assessed by validated screening tools in patient-facing healthcare professionals and non-patient-facing healthcare professional at baseline, phase 2 and phase 3.

Figure 3 compares the rates of probable mental health and burnout between patient-facing HCPs and non-patient-facing HCPs. At baseline and in the follow-up surveys, there was no considerable difference in the rates of probable major depressive disorder, generalised anxiety disorder and clinical insomnia among those who were patient-facing and those who were not. However, the rates of emotional exhaustion were considerably higher in patient-facing HCPs (42.7%) compared with non-patient-facing HCPs (33.9%) at baseline; this increased over both follow-up periods in patient-facing HCPs (43.1% at phase 2 and 44.6% at phase 3), but not non-patient-facing HCPs (37.4% at phase 2 and 32.4% at phase 3). The rates of depersonalisation were also considerably higher in patient-facing HCPs (14.4%) compared with non-patient-facing HCPs (6.7%) at baseline, and this increased at each follow-up phase in both patient-facing (15.5% at phase 2 and 21.6% at phase 3) and non-patient-facing HCPs (12.2% at phase 2 and 19.6% at phase 3). Regarding well-being, the proportion meeting the criteria for average-to-high well-being was relatively higher in patient-facing HCPs compared with non-patient-facing HCPs at baseline (75.5% *v*. 70.7%) and phase 3 (70.8% *v*. 64.1%). For specific rates of these mental health, burnout and well-being outcomes at each phase in patient-facing HCPs and non-patient-facing HCPs, see Supplementary Table 7.

### Adjusted mixed-effects linear regression models evaluating the risk of mental health conditions between HCPs and non-HCPs, and between patient-facing HCPs and non-patient-facing HCPs

**Fig. 4:**
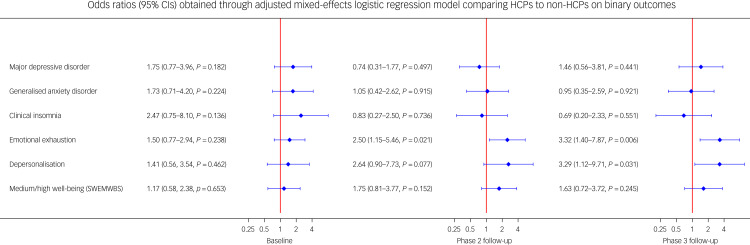
Separate mixed-effects logistic regression models calculating the odds for each outcome in HCPs compared with non-HCPs at baseline, phase 2 and phase 3. Blue plots denote risk (odds) with 95% confidence intervals for HCPs to meet criteria for outcomes, relative to non-HCPs (red line). The number of participants included in each regression model varied slightly for each outcome and for each phase (see Supplementary Table 6 for participant numbers with valid data for each outcome at each phase). HCP, healthcare professional; SWEMWBS, Short Warwick–Edinburgh Mental Wellbeing Scale.

Figure 4 shows the risk of probable mental health outcomes in HCPs compared with non-HCPs, after adjusting for other confounders. At baseline, compared with non-HCPs, there was no significant increase in the risk of the mental health, burnout and well-being outcomes in HCPs. However, in phase 2, HCPs (as compared with non-HCPs) had a 2.5-fold significantly increased risk of emotional exhaustion (adjusted odds ratio 2.50, 95% CI 1.15–5.46, *P* = 0.021), with no significant increased risk of other outcomes. At phase 3, the difference in risk between HCPs and non-HCPs on burnout domains further increased, with HCPs having more than 3.3-fold significantly increased risk of emotional exhaustion (odds ratio 3.32, 95% CI 1.40–7.87, *P* = 0.006) and depersonalisation (odds ratio 3.29, 95% CI 1.12–9.71, *P* = 0.031), with no differences in other outcomes (see Supplementary Figures 1 and 2 for adjusted differences in mean scores on the respective mental health, burnout and well-being measures at baseline, phase 2 and phase 3).

**Fig. 5:**
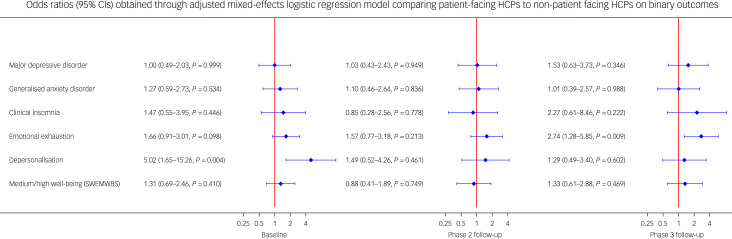
Separate mixed-effects logistic regression models calculating the odds for each outcome in patient-facing HCPs compared with non-patient-facing HCPs at baseline, phase 2 and phase 3. Blue plots denote risk (odds) with 95% confidence intervals for patient-facing HCPs to meet criteria for outcomes, relative to non-patient-facing HCPs (red line). The number of participants included in each regression model varied slightly for each outcome and for each phase (see Supplementary Table 7 for participant numbers with valid data for each outcome at each phase). HCP, healthcare professional; SWEMWBS, Short Warwick–Edinburgh Mental Wellbeing Scale.

At baseline, patient-facing HCPs had a five-fold increased risk of depersonalisation (odds ratio 5.02, 95% CI 1.65–15.26, *P* = 0.004) compared with non-patient-facing HCPs (Fig. 5), but no significant difference in the other outcomes. At phase 2 (Fig. 5), patient-facing HCPs had no significant increased risk of any mental health, burnout or well-being outcomes. However, at phase 3, although the difference in risk between patient-facing HCPs and non-patient-facing HCPs on the depersonalisation (burnout) domain had diminished, the difference in risk between the two groups on the emotional exhaustion domain had increased: patient-facing HCPs had a 2.7-fold increased risk of emotional exhaustion (odds ratio 2.74, 95% CI 1.28–5.85, *P* = 0.009), but no significant increased risk of other outcomes (see Supplementary Figures 3 and 4 for adjusted differences in mean scores on the respective mental health, burnout and well-being measures at baseline, phase 2 and phase 3).

## Discussion

To our knowledge, this is the first study examining the risk of probable mental health issues and burnout outcomes in HCPs compared with non-HCPs over multiple phases during the pandemic. This is also the first longitudinal study to differentiate between patient-facing HCPs and non-patient-facing HCPs. In this cohort study, both HCPs and non-HCPs had considerable rates of probable mental health issues during the COVID-19 pandemic. The rates of HCPs with probable major depressive disorder in HCPs reported here are similar to previous reports on UK HCPs during the pandemic;^27^ however, rates of probable generalised anxiety disorder are considerably lower in our study, which is likely explained by the different time points for data collection. We are unaware of normative data for these measures in the UK general population before the pandemic, but the observed rates for probable depression and anxiety in our study for HCPs and non-HCPs are in excess of pre-pandemic general population data elsewhere.^28,29^ Interestingly, there was no significant difference in the risk of these mental health conditions between HCPs and non-HCPs, contrary to other reports.^30^ On the other hand, compared with non-HCPs, there was a 3.3-fold increased risk of both emotional exhaustion and depersonalisation domains of burnout among HCPs by phase 3 follow-up. These findings not only suggest that HCPs are disproportionately affected on burnout domains, but show that within HCPs, patient-facing HCPs were at 2.7-fold increased risk of emotional exhaustion by phase 3 follow-up compared with non-patient-facing HCPs. Additionally, there is evidence here that the risk of emotional exhaustion in non-HCPs and non-patient-facing HCPs is reducing over time, whereas the risk of emotional exhaustion over time is increasing slightly in HCPs and patient-facing HCPs (this latter observation could be expected since the HCP group comprises primarily patient-facing HCPs). Because of the prolonged duration of the ongoing pandemic, our findings indicate serious concern that the high rates of burnout will persist or increase in HCPs (especially patient-facing HCPs), and result in a staffing and retention crisis facing healthcare policy makers.

### Strengths and limitations

This study has important strengths. First, we retained a good sample size across three distinct phases of assessment during the pandemic. Second, we used a wide array of validated mental health assessments to gain a comprehensive indicator of the mental health impact, as well as relatively underexamined issues such as burnout and insomnia.

However, there are a few, albeit minor, limitations that must be recognised. First, our burnout measure is a reduced version of the full Maslach Burnout Inventory scale. Although the items show good specificity when combined and used as a summative score, the two items alone may not capture the nuanced characteristics of burnout in our sample. Second, although the self-administered mental health screening tools are validated and appropriate for studying large samples, these could be less accurate than face-to-face psychiatric assessment and are instead indicators of probable mental health issues. Third, our non-HCP sample consists primarily of higher-education staff (with no involvement in healthcare setting), therefore non-HCPs in this study are a professional group, which might not reflect the general population in the UK. Indeed, both our HCP and non-HCP sample have relatively high levels of educational attainment, so it remains unclear from this analysis how the difference in risk of mental health issues and burnout relates to samples characterised by lower educational attainment. Fourth, as is the case with all self-reported cohort studies, our findings will be affected to some extent by volunteer bias: participants who are retained at phase 3 follow-up might not be wholly representative of the wider HCP and non-HCP population (indeed, baseline-only participants consisted of larger proportions of self-identified Asian ethnicity and male gender, and belonged to bigger households). As such, the findings are likely to be less generalisable to people of this demographic. Finally, because of the difference in sample sizes across each phase and slight overlap between samples at each phase, we are limited in what we can deduce regarding the trends of probable mental health issues and burnout over time in our study. To make valid interpretations of the trends in mental health outcomes over time, further longitudinal studies with consistent samples across multiple phases are required.

### Interpretation of findings

Our hypothesis, based on the perceived unique experiences of HCPs and in particular patient-facing HCPs during the initial phases of the COVID-19 pandemic, were largely not borne out in this study. HCPs and patient-facing HCPs did not show an increased risk of probable mental health conditions compared with non-HCPs and non-patient-facing HCPs, except for burnout. As such, interventions to mitigate the mental health impact of the pandemic should be addressed for the wider population, but additional tailored interventions to mitigate burnout are required for HCPs.

All HCPs (regardless of patient-facing status) are likely to experience increased exposure to workplace stressors (such as higher workloads and longer hours) during the pandemic, which can explain the differences in emotional exhaustion between HCPs and non-HCPs. Moreover, since the HCP group primarily consisted of patient-facing HCPs, the difference in emotional exhaustion could also be explained by the additive impact of facing patients^31^ and the relatively higher potential exposure to COVID-19 over time.^7^ Supporting this explanation, our baseline and follow-up data showed a considerable increase in positive COVID-19 tests in patient-facing HCPs compared with non-patient-facing HCPs.

The non-significant difference between the groups in increased risk of probable mental health outcomes (excluding burnout domains) also highlights the mental health burden of the pandemic on the wider population. For example, a previous study observed increasing psychological distress in the UK general population during the first lockdown restrictions, which declined to pre-pandemic levels by September 2020.^32^ Although we are limited in what we can deduce regarding the trends of these probable mental health outcomes over time because of the inconsistent sample sizes, we observed increased rates of probable major depressive disorder in HCPs and non-HCPs over the 4-month study period (which captured the entering of a second UK lockdown). This increase may reflect restrictions to coping mechanisms (e.g. leisure activities, socialising) during the second UK lockdown (October to December 2021).

Interestingly, the rates of probable generalised anxiety disorder increased across all phases for non-HCPs, whereas for HCPs it increased at phase 2, before declining markedly at phase 3 to near baseline levels. This increase in rates of generalised anxiety disorder for non-HCPs may reflect the increased uncertainty regarding entering and exiting lockdowns and the change in routines, and the anticipated increase in COVID-19 cases. However, the decline in generalised anxiety disorder rates from phase 2 to phase 3 in HCPs may reflect perceptions that COVID-19 cases will be controlled in response to the second UK lockdown.

An alternate explanation for the non-significant differences in mental health outcomes (excluding burnout) between our study groups might be explained by the duration of data collection. Perhaps collecting data over a longer duration would identify differences between HCP status and patient-facing status on the risk of depression, anxiety and insomnia. For example, some researchers have observed burnout to precede depression over a 3-year period and not *vice versa*.^33^ Indeed, burnout may also predict later insomnia.^34^

Aside from the potential onset of later mental health conditions, burnout is also prospectively associated with various cardiovascular and metabolic diseases.^35^ Therefore, effective interventions in the healthcare sector are urgently needed to mitigate burnout and its physical and psychological consequences. One strategy for HCPs and non-HCPs could be managerial/workplace/organisational support: analyses evaluating the association between perceived managerial/workplace/organisational support and mental health scores over time in HCPs and non-HCPs are ongoing.^21^

In conclusion, our cohort study demonstrates that there was a significant mental health toll on both HCPs and non-HCPs during the COVID-19 pandemic; however, compared with non-HCPs, there was a significantly higher risk of burnout among HCPs, with this difference increasing over follow-up. Furthermore, patient-facing HCPs may also be at increased risk of burnout (specifically emotional exhaustion) compared with non-patient-facing HCPs. Longer-term follow-up is required to evaluate whether the risk of other mental health outcomes differ at later time points during the COVID-19 pandemic, and to examine the potential impact of burnout on the physical health of HCPs. Further follow-up will inform resource-planning and health policy decisions.

## Data Availability

Anonymised data, the data dictionary and survey materials are available from the corresponding author, A.G., on request. The study protocol is available at https://doi.org/10.3389/fpsyg.2021.616280.

## Supplementary material

For supplementary material accompanying this paper visit https://doi.org/10.1192/bjo.2022.579. click here to view supplementary material
