# Supplementary material for: Longitudinal comparisons of mental health, burnout and well-being in patient-facing, non-patient-facing healthcare professionals and non-healthcare professionals during the COVID-19 pandemic: findings from the CoPE-HCP study
Source: BJPsych Open. 2022 Sep 27;8(5):e173. doi: 10.1192/bjo.2022.579 (PMC9530379; doi:10.1192/bjo.2022.579)

| **Supplemental Table 1.** Chi square analysis of characteristics of baseline-only (n = 666) and rest of participants involved at other phases (n = 1055). (Total N = 1721). | | | | |
| --- | --- | --- | --- | --- |
|  | Response | Baseline-only (n, 666) (%) | Rest (n, 1055) (%) | Chi squared result |
| **Age** | 18-25 years | 33 (5.0) | 51 (4.8) | 3.8, *p* = 0.58 |
|  | 26-35 years | 169 (25.4) | 270 (25.6) |  |
|  | 36-50 years | 275 (41.3) | 419 (39.7) |  |
|  | 51-60 years | 155 (23.3) | 242 (22.9) |  |
|  | 61-70 years | 33 (5.0) | 66 (6.3) |  |
|  | > 70 years | 1 (0.2) | 7 (0.7) |  |
| **Ethnicity** | White | 324 (48.7) | 812 (77.0) | 153.9, *p <* 0.001 |
|  | Asian | 238 (35.7) | 148 (13.8) |  |
|  | Black | 40 (6.0) | 38 (3.6) |  |
|  | Mixed | 25 (3.8) | 26 (2.5) |  |
|  | Other | 20 (3.0) | 22 (2.1) |  |
|  | Prefer not to say | 19 (2.9) | 11 (1.0) |  |
| **Gender identity** | Female | 432 (64.9) | 782 (74.1) | 23.4, *p* < 0.001 |
|  | Male | 217 (32.6) | 266 (25.2) |  |
|  | Prefer not to say | 11 (1.7) | 5 (0.5) |  |
|  | Prefer to self-define | 6 (0.9) | 2 (0.2) |  |
| **Relationship status** | Divorced | 24 (3.6) | 32 (3.0) | 5.2, *p* = 0.27 |
|  | Prefer not to say | 24 (3.6) | 25 (2.4) |  |
|  | Married/Living w/ partner or family | 443 (66.5) | 698 (66.2) |  |
|  | Other | 77 (4.1) | 33 (3.1) |  |
|  | Single | 148 (22.2) | 267 (25.3) |  |
| **Number living in household** | 1 | 87 (13.1) | 140 (13.3) | 20.4, *p* < 0.001 |
|  | 2 | 187 (28.1) | 355 (33.7) |  |
|  | 3-5 | 341 (51.2) | 526 (49.9) |  |
|  | 6 or more | 51 (7.7) | 34 (3.2) |  |
| **Highest level of education** | GCSEs / A-levels | 44 (6.6) | 77 (7.3) |  |
|  | Bachelor’s / diploma | 292 (43.8) | 479 (45.4) |  |
|  | Master's / PhD | 281 (42.2) | 434 (41.1) |  |
|  | Other | 49 (7.4) | 65 (6.2) |  |
| **Mental health outcomes at baseline*** | Major depressive disorder | 121 (21.7) | 262 (25.7) | 3.2 *p* = 0.07 |
|  | Generalised anxiety disorder | 101 (18.3) | 207 (20.3) | 1.0, *p* = 0.32 |
|  | Clinical insomnia | 72 (13.2) | 172 (17.0) | 3.8, *p* = 0.052 |
|  | Emotional exhaustion | 226 (43.6) | 409 (40.6) | 1.2, *p* = 0.27 |
|  | Depersonalisation | 76 (14.6) | 127 (12.6) | 1.2, *p* = 0.27 |
|  | High-medium wellbeing | 396 (75.6) | 750 (74.3) | 0.3, *p* = 0.60 |
| *Note.* All demographic data is self-reported. ‘Asian’ category includes South Asian, Chinese, and any other Asian background. ‘Mixed’ category includes mixed Black and White, mixed Asian and White, and mixed any other/multiple ethnic backgrounds. | | | | |

| **Supplemental Table 2.** Baseline demographic, social and educational characteristics of participants, categorised as HCPs and non-HCPs. | | | | |
| --- | --- | --- | --- | --- |
| **Characteristic** | **Response** | **HCPs**  **n = 1574 (%)** | **Non-HCPs**  **n = 147 (%)** | **Overall**  **N = 1721 (%)** |
| **Age** | 18-25 years | 76 (4.8) | 8 (5.4) | 84 (4.9) |
|  | 26-35 years | 390 (24.8) | 49 (33.3) | 439 (25.5) |
|  | 36-50 years | 638 (40.5) | 56 (38.1) | 694 (40.3) |
|  | 51-60 years | 372 (23.6) | 25 (17.0) | 397 (23.1) |
|  | 61-70 years | 92 (5.8) | 7 (4.8) | 99 (5.8) |
|  | > 70 years | 6 (0.4) | 2 (1.4) | 8 (0.5) |
| **Ethnicity** | White | 1027 (65.3) | 109 (74.2) | 1136 (66.0) |
|  | Asian | 359 (22.8) | 25 (17.0) | 384 (22.3) |
|  | Black | 74 (4.7) | 4 (2.7) | 78 (4.5) |
|  | Mixed | 48 (3.1) | 3 (2.0) | 51 (3.0) |
|  | Other | 39 (2.5) | 3 (2.0) | 42 (2.4) |
|  | Prefer not to say | 27 (1.7) | 3 (2.0) | 30 (1.7) |
| **Gender identity** | Female | 1105 (70.2) | 109 (74.2) | 1214 (70.5) |
|  | Male | 447 (28.4) | 36 (24.5) | 483 (28.1) |
|  | Prefer not to say | 14 (0.9) | 2 (1.4) | 16 (0.9) |
|  | Prefer to self-define | 8 (0.5) | 0 (0.0) | 8 (0.5) |
| **Relationship status** | Divorced | 54 (3.4) | 2 (1.4) | 56 (3.3) |
|  | I prefer not to disclose | 46 (2.9) | 3 (2.0) | 49 (2.9) |
|  | Married/Living with partner or family | 1048 (66.6) | 93 (63.3) | 1141 (66.3) |
|  | Other (please specify) | 52 (3.3) | 8 (5.4) | 60 (3.4) |
|  | Single | 374 (23.8) | 41 (27.9) | 415 (24.1) |
| **Number living in household** | 1 | 210 (13.3) | 17 (11.6) | 227 (13.2) |
|  | 2 | 487 (30.9) | 55 (37.4) | 542 (31.5) |
|  | 3-5 | 799 (50.8) | 68 (46.3) | 867 (50.4) |
|  | 6 or more | 78 (5.0) | 7 (4.8) | 85 (4.9) |
| **Highest level of education** | GCSEs or A-levels | 113 (7.2) | 8 (5.4) | 121 (7.0) |
|  | Bachelor’s degree/diploma | 735 (46.7) | 36 (24.5) | 771 (44.8) |
|  | Master's degree or PhD | 613 (39.0) | 102 (69.4) | 715 (41.6) |
|  | Other | 113 (7.2) | 1 (0.7) | 114 (6.6) |
| Other education generally includes professional qualifications such as Doctor of Medicine, MBA, | | | | |

| **Supplemental Table 3.** Baseline demographic, social and educational characteristics of all HCPs, categorised into patient facing and non-patient facing HCPs. | | | | |
| --- | --- | --- | --- | --- |
| **Characteristic** | **Response** | **Patient facing HCPs**  **n = 1345 (%)** | **Non-patient facing HCPs**  **n = 192 (%)** | **Overall**  **N = 1537 (%)** |
| **Age** | 18-25 years | 64 (4.8) | 9 (4.7) | 73 (4.8) |
|  | 26-35 years | 346 (25.7) | 35 (18.2) | 381 (24.8) |
|  | 36-50 years | 561 (41.7) | 66 (34.4) | 627 (40.8) |
|  | 51-60 years | 301 (22.4) | 60 (31.3) | 361 (23.5) |
|  | 61-70 years | 70 (5.2) | 19 (9.9) | 89 (5.8) |
|  | > 70 years | 3 (0.2) | 3 (1.6) | 6 (0.4) |
| **Ethnicity** | White | 865 (64.3) | 140 (72.9) | 1005 (65.4) |
|  | Asian | 313 (23.3) | 37 (19.3) | 350 (22.8) |
|  | Black | 66 (4.9) | 6 (3.1) | 72 (4.7) |
|  | Mixed | 42 (3.1) | 5 (2.6) | 47 (3.1) |
|  | Other | 35 (2.6) | 3 (1.6) | 38 (2.5) |
|  | Prefer not to say | 24 (1.8) | 1 (0.5) | 25 (1.6) |
| **Gender identity** | Female | 945 (70.3) | 136 (70.8) | 1081 (70.3) |
|  | Male | 382 (28.4) | 53 (27.6) | 435 (28.3) |
|  | Prefer not to say | 11 (0.8) | 2 (1.0) | 13 (0.9) |
|  | Prefer to self-define | 7 (0.5) | 1 (0.5) | 8 (0.5) |
| **Relationship status** | Divorced | 43 (3.2) | 9 (4.7) | 52 (3.4) |
|  | I prefer not to disclose | 36 (2.7) | 5 (2.6) | 41 (2.7) |
|  | Married/Living with partner or family | 896 (66.6) | 129 (67.2) | 1025 (66.7) |
|  | Other (please specify) | 42 (3.1) | 7 (3.7) | 49 (3.2) |
|  | Single | 328 (24.4) | 42 (21.9) | 370 (24.1) |
| **Number living in household** | 1 | 181 (13.5) | 23 (12.0) | 204 (13.3) |
|  | 2 | 408 (30.3) | 66 (34.4) | 474 (30.8) |
|  | 3-5 | 686 (51.0) | 97 (50.5) | 783 (50.9) |
|  | 6 or more | 70 (5.2) | 6 (3.1) | 76 (4.9) |
| **Highest level of education** | GCSEs or A-levels | 69 (5.1) | 38 (19.8) | 107 (7.0) |
|  | Bachelor’s degree/diploma | 657 (48.9) | 65 (33.9) | 722 (47.0) |
|  | Master's degree or PhD | 522 (38.8) | 78 (40.6) | 600 (39.0) |
|  | Other | 97 (7.2) | 11 (5.7) | 108 (7.0) |
| Other education includes professional qualifications such as Doctor of Medicine, MBA, | | | | |

| **Supplementary Table 4**. Self-reported positive COVID-19 test result at baseline and phase 3 follow-up, stratified by HCP status. | | | | |
| --- | --- | --- | --- | --- |
|  |  | HCPs (%) | Non-HCPs (%) | Overall (%) |
| Baseline (n, 1597) |  | 269 (18.5) | 4 (2.8) | 273 (17.1) |
| Phase 3 follow-up (n, 733) |  | 178 (26.5) | 12 (20.0) | 190 (25.9) |
| *Note.* The total number of HCPs providing adequate responses on this item was 1455 at baseline, and 673 at phase 3 follow-up. The total number of non-HCPs providing adequate responses on this item was 142 at baseline, and 60 at phase 3 follow-up. | | | | |

| **Supplementary Table 5**. Self-reported positive COVID-19 test result at baseline and phase 3 follow-up in HCPs, stratified by patient facing status. | | | | |
| --- | --- | --- | --- | --- |
|  |  | Patient facing HCPs (%) | Non-patient facing HCPs (%) | Overall (%) |
| Baseline (n, 1455) |  | 243 (19.1) | 26 (14.1) | 269 (18.5) |
| Phase 3 follow-up (n, 736) |  | 164 (26.0) | 15 (14.4) | 179 (24.3) |
| *Note.* The total number of patient facing HCPs providing adequate responses on this item was 1271 at baseline, and 632 at phase 3 follow-up. The total number of non-patient facing HCPs providing adequate responses on this item was 184 at baseline, and 104 at phase 3 follow-up. | | | | |

| **Supplementary Table 6.** Prevalence of mental health, burnout, and wellbeing outcomes in HCPs and non-HCPs at baseline, phase 2, and phase 3. | | | |
| --- | --- | --- | --- |
|  |  | % of HCPs | % of non-HCPs |
| Major depressive disorder |  |  |  |
|  | Baseline | 24.7 | 20.4 |
|  | Phase 2 | 24.6 | 27.4 |
|  | Phase 3 | 28.0 | 26.2 |
| Generalised anxiety disorder |  |  |  |
|  | Baseline | 19.9 | 16.9 |
|  | Phase 2 | 23.5 | 24.5 |
|  | Phase 3 | 20.8 | 25.0 |
| Clinical insomnia |  |  |  |
|  | Baseline | 16.1 | 11.4 |
|  | Phase 2 | 16.9 | 17.0 |
|  | Phase 3 | 16.3 | 15.5 |
| Emotional exhaustion (burnout) |  |  |  |
|  | Baseline | 41.9 | 39.3 |
|  | Phase 2 | 42.8 | 35.2 |
|  | Phase 3 | 43.2 | 35.4 |
| Depersonalisation (burnout) |  |  |  |
|  | Baseline | 13.4 | 12.1 |
|  | Phase 2 | 15.5 | 11.4 |
|  | Phase 3 | 21.2 | 15.9 |
| Average-high wellbeing |  |  |  |
|  | Baseline | 75.0 | 72.9 |
|  | Phase 2 | 70.8 | 65.7 |
|  | Phase 3 | 70.0 | 64.3 |

*Note.* The number of HCPs and non-HCPs providing valid data differs for each mental health outcome: for major depressive disorder, 1434 HCPs and 142 non-HCPs were included at baseline, followed by 836 and 106 at phase 2, and 724 and 84 at phase 3 follow-up. For generalised anxiety disorder, 1429 HCPs and 142 non-HCPs were included at baseline, followed by 834 and 106 at phase 2, and 723 and 84 at phase 3 follow-up. For clinical insomnia, 1418 HCPs and 141 non-HCPs were included at baseline, followed by 834 and 106 at phase 2, and 722 and 84 at phase 3 follow-up. For emotional exhaustion and depersonalisation, 1386 HCPs and 140 non-HCPs were included at baseline, followed by 828 and 105 at phase 2, and 717 and 82 at phase 3 follow-up. For wellbeing, 1393 HCPs and 140 non-HCPs were included at baseline, followed by 828 and 105 at phase 2, and 720 and 84 at phase 3 follow-up.

| **Supplementary Table 6.** Prevalence of mental health, burnout, and wellbeing outcomes in patient facing HCPs and non-patient facing HCPs at baseline, phase 2, and phase 3. | | | |
| --- | --- | --- | --- |
|  |  | % of patient facing HCPs | % of non-patient facing HCPs |
| Major depressive disorder |  |  |  |
|  | Baseline | 24.5 | 25.7 |
|  | Phase 2 | 24.3 | 27.0 |
|  | Phase 3 | 27.7 | 26.9 |
| Generalised anxiety disorder |  |  |  |
|  | Baseline | 20.2 | 19.8 |
|  | Phase 2 | 23.6 | 24.4 |
|  | Phase 3 | 20.4 | 22.3 |
| Clinical insomnia |  |  |  |
|  | Baseline | 16.3 | 14.7 |
|  | Phase 2 | 16.5 | 18.3 |
|  | Phase 3 | 16.4 | 15.5 |
| Emotional exhaustion (burnout) |  |  |  |
|  | Baseline | 42.7 | 33.9 |
|  | Phase 2 | 43.1 | 37.4 |
|  | Phase 3 | 44.6 | 32.4 |
| Depersonalisation (burnout) |  |  |  |
|  | Baseline | 14.4 | 6.7 |
|  | Phase 2 | 15.5 | 12.2 |
|  | Phase 3 | 21.6 | 19.6 |
| Average-high wellbeing |  |  |  |
|  | Baseline | 75.5 | 70.7 |
|  | Phase 2 | 70.8 | 71.3 |
|  | Phase 3 | 70.8 | 64.1 |

*Note.* The number of patient facing HCPs and non-patient facing HCPs providing valid data differs for each mental health outcome: for major depressive disorder, 1275 patient facing HCPs and 187 non-patient facing HCPs were included at baseline, followed by 729 and 115 at phase 2, and 629 and 104 at phase 3 follow-up. For generalised anxiety disorder, 1269 patient facing HCPs and 187 non-patient facing HCPs were included at baseline, followed by 728 and 115 at phase 2, and 629 and 103 at phase 3 follow-up. For clinical insomnia, 1261 patient facing HCPs and 184 non-patient facing HCPs were included at baseline, followed by 728 and 115 at phase 2, and 628 and 103 at phase 3 follow-up. For emotional exhaustion and depersonalisation, 1232 patient facing HCPs and 180 non-patient facing HCPs were included at baseline, followed by 722 and 115 at phase 2, and 624 and 102 at phase 3 follow-up. For wellbeing, 1238 patient facing HCPs and 181 non-patient facing HCPs were included at baseline, followed by 722 and 115 at phase 2, and 626 and 103 at phase 3 follow-up.

**Supplementary Fig. 1.** Adjusted difference in mean scores for depression (PHQ-9), anxiety (GAD-7), clinical insomnia (ISI-7), and burnout summative scores (EEDP2Q) in HCPs and non-HCPs at baseline, phase 2, and phase 3.


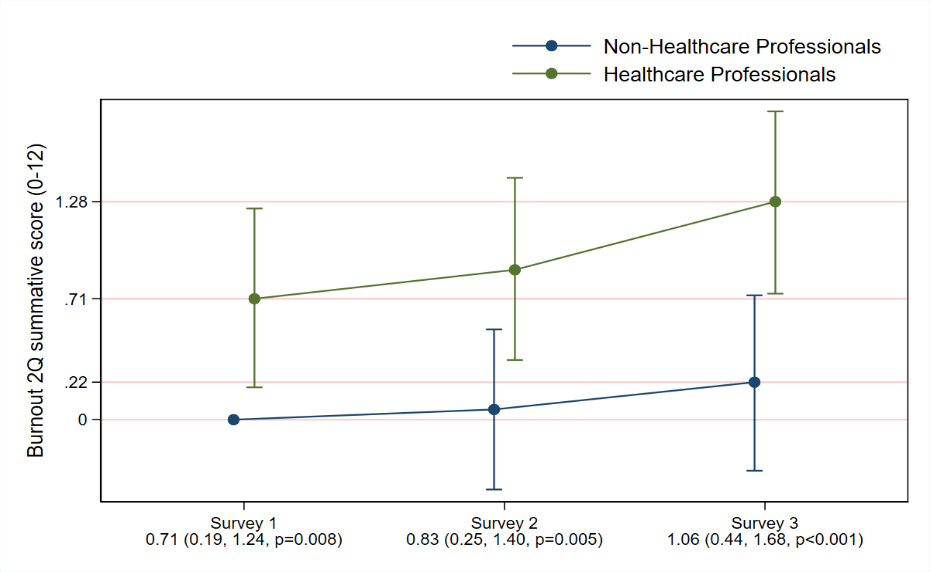

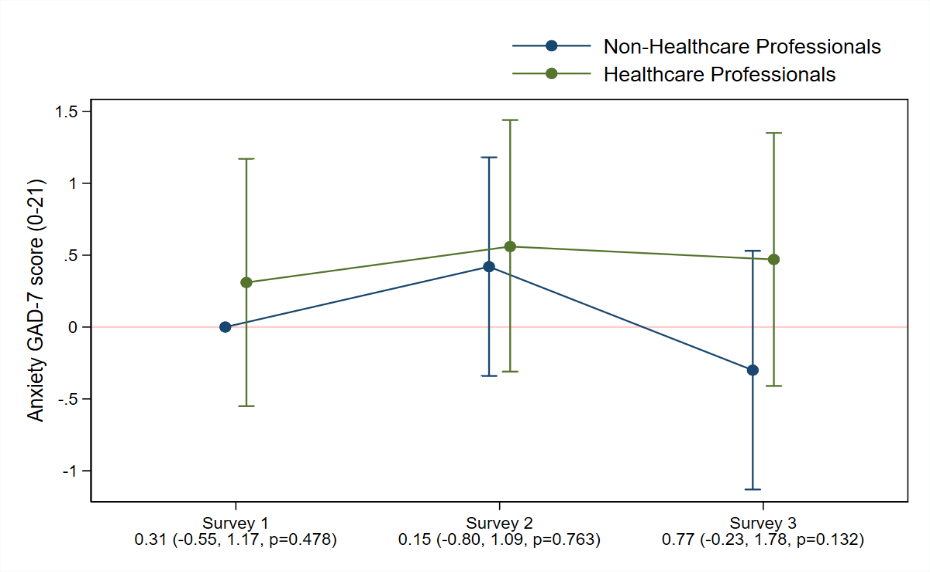

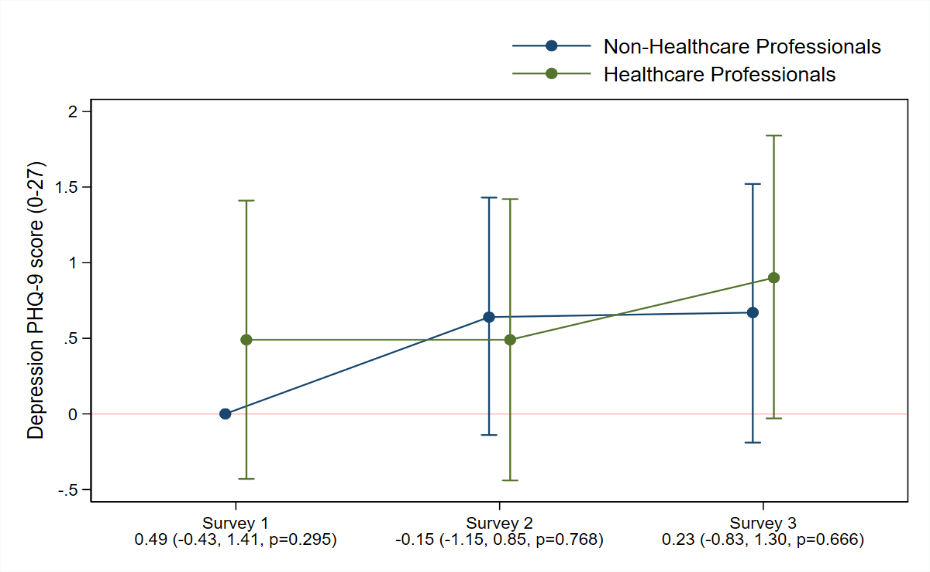

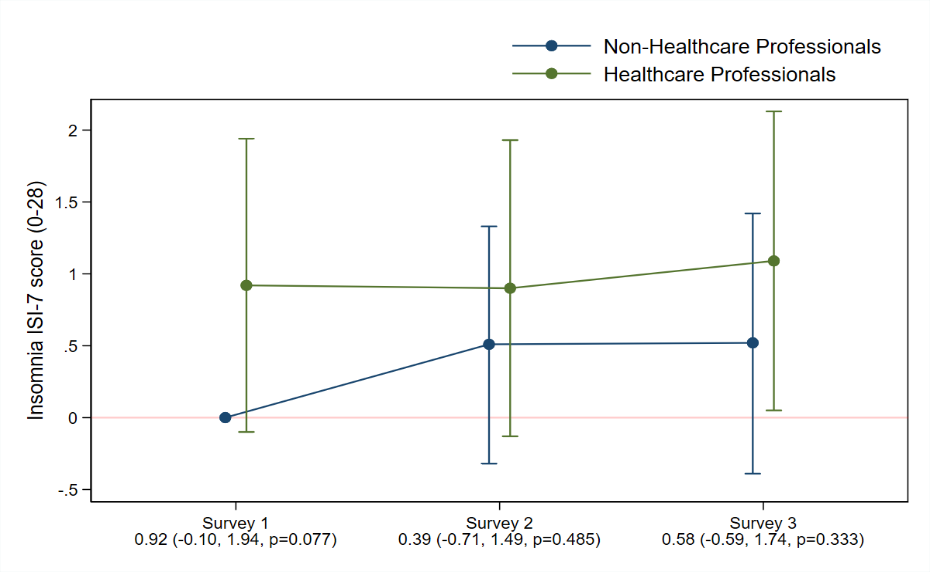


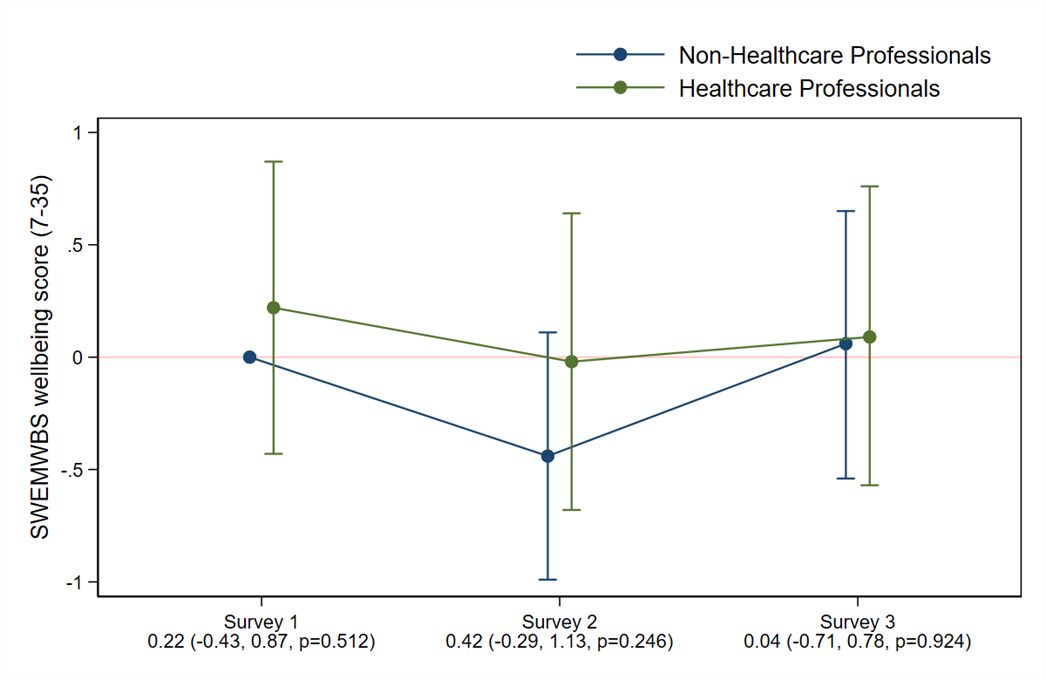
**Supplementary Fig. 2.** Adjusted difference in mean scores for wellbeing (SWEMWBS) in HCPs and non-HCPs at baseline, phase 2, and phase 3.

**Supplementary Fig. 3.** Adjusted difference in mean scores for depression (PHQ-9), anxiety (GAD-7), clinical insomnia (ISI-7), and burnout summative scores (EEDP2Q) in patient facing HCPs and non-patient facing HCPs at baseline, phase 2, and phase 3.


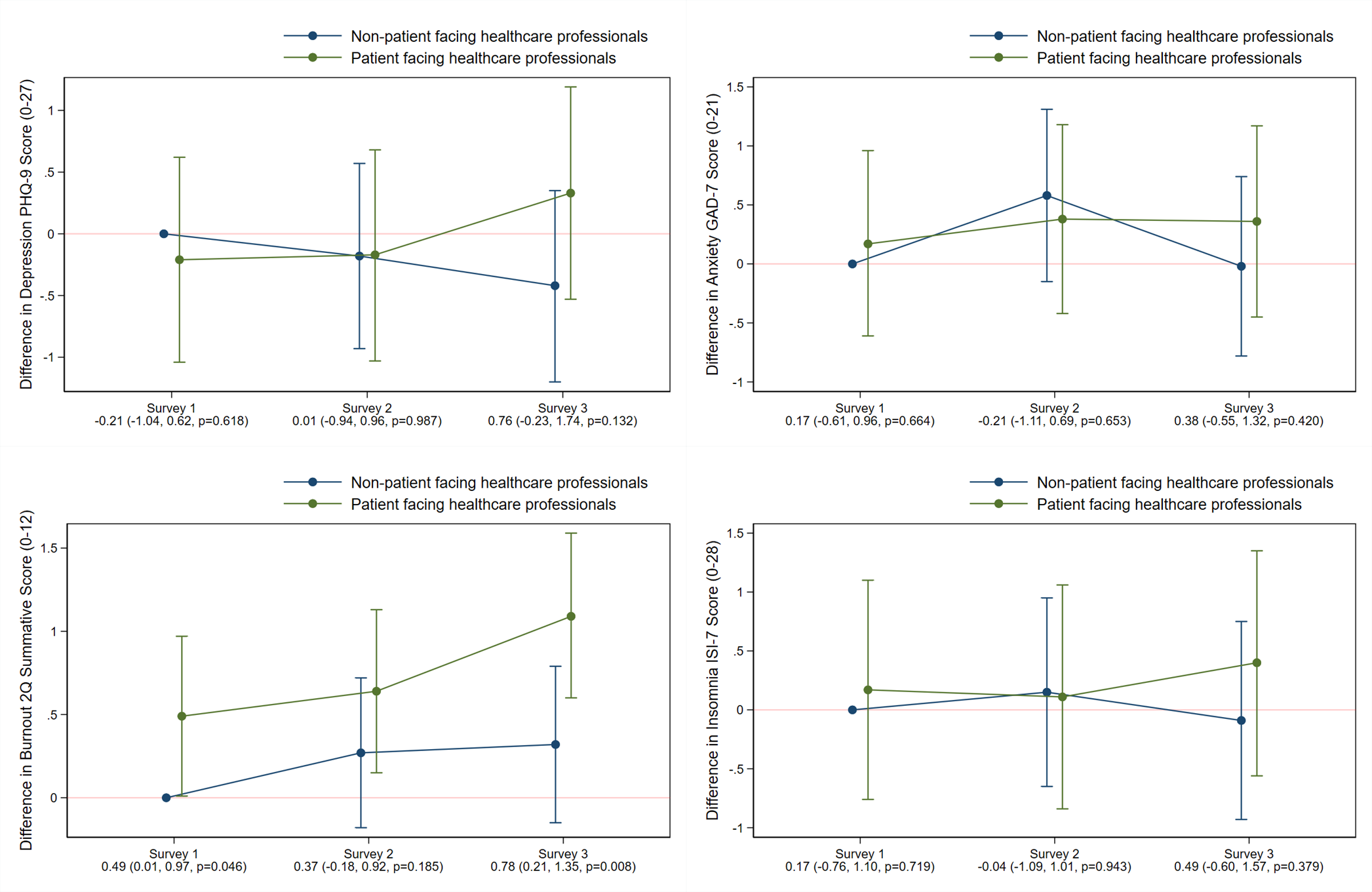


**Supplementary Fig. 4.** Adjusted difference in mean scores for wellbeing (SWEMWBS) in patient facing HCPs and non-patient facing HCPs at baseline, phase 2, and phase 3.


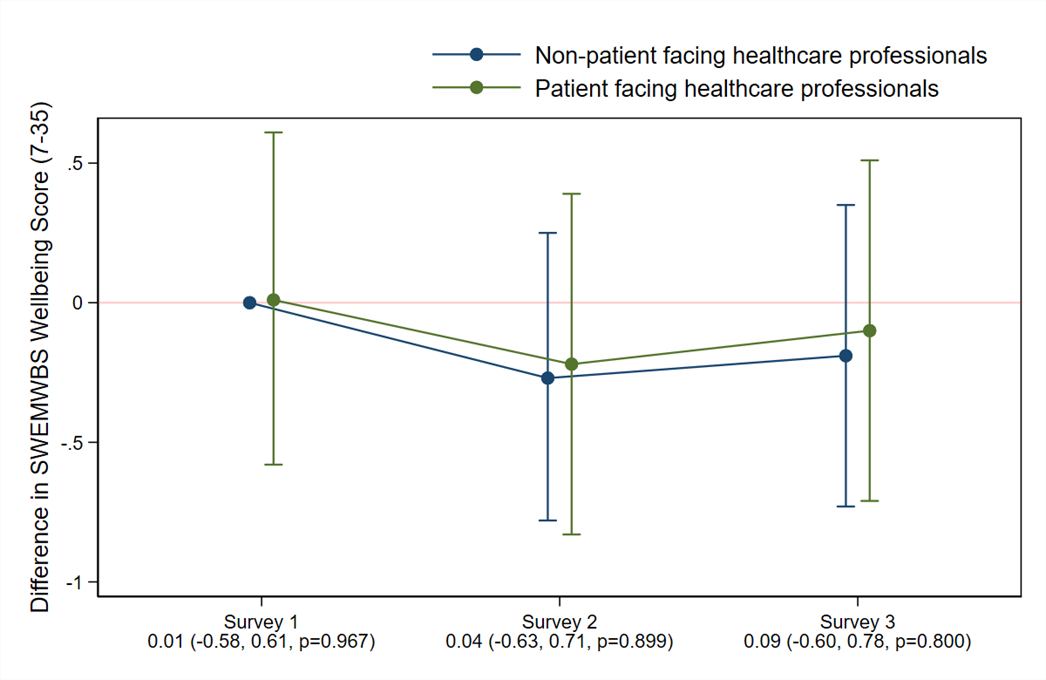

Supplement: Supplementary file 1 [file bjosup.zip › S2056472422005798sup001.docx]
